# Ohio State Dental Society

**Published:** 1885-12

**Authors:** E. G. Betty


(TO BE CONTINUED.)

### Proceedings and Discussions of the Ohio State Dental Society, Re-organized at Chillicothe, Ohio, October 28th, 29th and 30th, 1885.

REPORTED BY E. G. BETTY, D.D.S.

The first annual meeting of this Society was called to order at 11 o'clock Wednesday morning in the Council Chamber at the City Buildings, President C. H. James in the Chair.

Prayer was offered by the Chaplain, Rev. Dr. Chas. Fisher, Rector of St. Paul's Episcopal Church.

The minutes of the preliminary organization at Columbus, in October, 1884, were read and approved.

President James then delivered a short address, in which he said: "I wish to express my thanks to the members of the Society for the honor they have conferred upon me in making me their presiding officer. My object and desire is to leave no regret upon your part for having intrusted to me such an important duty, and on mine, that I have neglected no duty or labor. I wish especially to call your attention to the necessity of a thorough organization of the Ohio State Dental Society, and to that end you will forgive my relation of a few historical facts connected with the old Society.

In May, 1866, invitations were issued to the Profession throughout our State to meet at Columbus and organize a State Dental Society. The appeal was heartily and enthusiastically responded to, and on the 26th of June, of that year, forty-eight names were enrolled upon the books. This list has continually been augmented from year to year, until the total of 227 was reached in 1884.

One of the bad features of the Society was, that it was burthened with too many rules, laws, etc., and too much time was frittered away in useless wrangles to the exclusion of the legitimate professional business, which the members met together to discuss.

Something had to be done to remedy this condition of affairs, and to re-awaken the interest of the Profession, and to divide the burden born by the willing few, amongst a large number. He who does not associate himself with others, travels in a rut, with the result of lack of interest, ambition, and professional progress. By mutual exchange of thoughts and methods, we work out a professional good in uniting forces not otherwise of much avail, in this way attaining power and consequent advantage to the Society and all its members. We want to build a permanent Society—a grand one, and bequeath it to our successors as a permanent organization of grandeur, strength, and power for all time. Lastly, we want to frame a State law that will be thorough in all respects, and operate to the ultimate honor and good of all."

Dr. F. H. Rehwinkel, Chairman of the Executive Committee, moved that the consideration of the Articles of the Constitution and By-Laws, be made a special order of business for 2 o'clock and at 8 o'clock p. m. the consideration of the Dental Law, be also made a special order of business, inasmuch as the representative elect from Ross County, Col. Entreken, will be present with us to assist us with his advice as to the best method of proceeding to get the bill framed and brought before the legislature. Carried.

I also have to announce, Mr. President, that Dr. Boedecker, of New York, cannot meet with us as we anticipated. He was obliged to go to Europe to consult a specialist for the treatment of a member of his family. Herr Erbst was also expected by Dr. Boedecker to come over with him. I have a number of specimens of his work which I shall be pleased to have the members consider as their own property, to examine them and burst them open if they wish, in order to see the adaptation of the gold to the walls of the cavity."

On resolution of Dr. J. Taft, the hours of the sessions were fixed at 9 to 12 m., 1:30 to 5:30 p. m., and from 7:30 till adjournment. A motion was carried to devote the hours between 1:30 and 3 o'clock p. m. Thursday, to the exhibition of instruments, appliances, etc.

Dr. Taft. The organization at present consists of the eighteen charter members only, but, there are many others present, whom it would be desirable to have take part in the organization, as they are interested in this matter as well as anyone else. Any others who wish to join can do so in the ordinary way after the organization is completed. Seconded.

Dr. H. A. Smith. I think we ought to go slow in this matter, as the incorporators only have a right to adopt Articles of the Constitution, and we would not gain anything by inviting them to participate until the organization has been perfected.

Dr. G. Watt. Upon mature thought, I do not think the resolution would be legal under the Articles of Incorporation. I would amend the resolution by inviting them to participate in the discussion, though they are not entitled to vote.

Dr. Taft. I have re-arranged the resolution to include the points made by Drs. Smith and Watt, and it now reads: *Resolved*, That those members who are present and were in good standing at the dissolution of the old Society, be cordially invited to participate in the discussion of the organization at this time, as regards its Constitution, By-Laws, etc. Also, that visitors from other State Societies present, be invited to the same privilege. The resolution was seconded and carried.

A draft of the Constitution and By-Laws made by Dr. Rehwinkel was read by him.

Dr. Taft, Chairman Committee on Voluntary Essays, reported a paper by Mr. Beer, of Newcastle on Tyne, England, and one by Dr. Parsons, of Savannah, Georgia. Adjourned.

### FIRST DAY--AFTERNOON SESSION.

The meeting was called to order by President James at 2:20. Secretary Callahan read the minutes of the morning session, and no objection being offered, they stood approved.

On motion of Dr. J. Taft, Dr. H. H. Harrison read a draft for a Constitution, made up mainly from that of the old Society, together with such changes and additions, as in his judgment suggested a betterment of the interests of the Society. Dr. Harrison stated that he did not wish to offer his draft, as he thought Dr. Rehwinkel's so fully covered the ground that others were not necessary.

The Society then considered the separate articles of Dr. Rehwinkel's draft, and with the exception of Section 3, of Article I. and II., of the Constitution, and Section 6, of Article I., of the By-Laws, was adopted; these Sections being referred back to the Committee to be changed. Adjourned.

### FIRST DAY--EVENING SESSION.

The Society came to order at 8 o'clock, when the minutes of the previous session were read and approved.

The consideration of the Dental Law was taken up as a special order of business and discussed at considerable length. Colonel Entreken, the Representative elect from Ross County, was present and materially assisted with his advice and counsel.

On resolution of Dr. Rehwinkel, the following Committee on Dental Law was appointed :

C. H. James, Cincinnati.
F. H. Rehwinkel, Chillicothe.
A. F. Emminger, Columbus.
J. R. Callahan, Hillsboro.
F. C. Runyan, Springfield.
J. E. Robinson, Cleveland.
C. H. Harroun, Toledo.
H. H. Harrison, Cadiz.
H. A. Smith, Cincinnati.

A standing vote of thanks was tendered Colonel Entreken. Adjourned.

SECOND DAY--MORNING SESSION.

At half-past nine o'clock, President James called the meeting to order, when the minutes of the first day's session were read and approved. The Constitution and By-Laws as follows were adopted as a whole, by an unanimous vote :

### CONSTITUTION.

### Article I.

Name. This Association shall be known as the Ohio State Dental Society.

### Article II.

The aims and objects are as follows: The elevation of the standard of Professional Education ; the advancement and cultivation of Dental Science and Literature, and the protection of the public from the evils of empiricism ; the promotion of the honor, usefulness and interests of the profession, and mutual fellowship and good feeling.

### Article III.

Section. 1. The membership of this Society shall consist of two classes, namely : Active and Honorary.

SEC. 2. *Active Members.* Any honorable practitioner of dentistry in the State of Ohio, who is a graduate of a reputable dental college, or who is in possession of a certificate of qualification from the Ohio State Board of Dental Examiners, may become an active member of this Society.

SEC. 3. *Honorary Membership.* Active members who have retired from practice and reputable practitioners of dentistry from other States or foreign countries, who are distinguished for their scientific and professional attainments, and by their labors on behalf of Dental Science.

SEC. 4. Any one eligible to active membership, shall not be proposed for honorary membership.

### ARTICLE IV.

#### OFFICERS.

SECTION 1. The officers of this Society shall consist of a President, two Vice-Presidents, a Recording Secretary and a Treasurer.

SEC. 2. *Board of Directors.* The management of this Society shall be by a Board, consisting of twelve active members and the officers of the Society as named in Section 1, of this Article, the whole number of the Board to be seventeen.

SEC. 3. *Board of Examiners.* A Board of Examiners consisting of five active members shall be elected annually, to serve for three years or until their successors are chosen.

### ARTICLE V.

SECTION 1. *Election of Officers.* The officers named in Section 1 of Article IV. shall be elected annually by ballot at a regular meeting. A majority of all votes cast shall be necessary to a choice.

SEC. 2. The Board of Directors shall be elected annually by ballot, at a regular meeting. A majority of all votes cast shall be necessary to a choice. They shall be divided into three classes of four each; the first class shall serve one year, the second class shall serve two years, the third class shall serve three years, and thereafter the successors for each class shall be elected for three years.

The officers of the Society as named in Section 1 of Article IV., shall be the officers of this Board.

SEC. 3. *Board of Examiners.* This Board shall consist of five active members, and shall be divided into three classes; the first class, consisting of one, shall be elected for one year; the second class, consisting of two, shall be elected for two years; the third class, consisting of two, shall be elected for three years.

A majority of all votes cast shall be necessary for a choice. They shall serve for one, two and three years, or until their successors shall have been chosen.

### ARTICLE VI.

### ELECTION OF MEMBERS.

SECTION 1. All elections shall be by ballot.

SEC 2. The applicants for membership shall state on printed form, their names in full, place of residence, post office address, County and State; whether they are graduates, and from what College or University; name the titles if they have any; if licensed, by what authority; how long they have been in continual practice and where.

SEC. 3. All applications for membership shall be presented by a member of this Society to the Committee on Membership, and if the applicant be reported favorably through the Board of Directors, he or she may be elected; four-fifths of all the votes cast shall be necessary to elect.

### ARTICLE VII.

### STANDING COMMITTEE.

The Board of Directors shall annually appoint the following standing committees, each one shall consist of three members, they may be chosen from the Board or otherwise:

1. A Committee of Arrangements.
2. A Committee on Publication and Voluntary Essays.
3. A Committee on Membership.

They shall respectively discharge the duties designated in the By-Laws.

### ARTICLE VIII.

### AMENDMENTS.

All proposed changes in, or amendments to this Constitution, shall be in writing, presented at a regular annual meeting, signed by five active members, and such alterations or amendments be noted in the announcements of the next regular meeting, and shall not be acted upon until that time and then adopted by only three-fourths of the votes of the active members present.

### BY-LAWS.

### ARTICLE I.

SECTION 1. *President.* The President shall preside at all meetings of the Society and Board of Directors. He shall give a deciding vote in all cases of a tie except in election of officers; call special meetings upon the written request of nine members of the Board of Directors, and perform such other duties as may from time to time be assigned to him.

SEC. 2. *First Vice-President.* In the absence of the President, he shall preside at the meetings of this Society and Board of Directors, and perform all other duties incumbent upon the President.

SEC. 3. *Second Vice-President.* In the absence of both President and First Vice-President, he shall preside at the meeting of the Society and Board of Directors, and shall perform all other duties pertaining to the office of President.

SEC. 4. *Recording Secretary.* The Recording Secretary shall keep an accurate record of the proceedings of the meetings of the Society; issue announcements and notices of meetings, and have the custody of the seal of the Society. He shall take charge of all letters and communications addressed to the Society, and shall conduct the correspondence as may be directed by the Board of Directors. He shall notify all officers of their election, committees of their appointments and the subjects referred to them; keep the manuscript, Constitution and By-Laws and Code of Ethics of the Society. He shall also keep a record of the membership, including resignations and other changes, and perform such other duties as may from time to time be assigned him.

SEC. 5. *Treasurer.* The Treasurer shall collect all dues, keep an exact account of all receipts and expenditures and the vouchers therefor; notify all members of arrearages, and present a written report of the conduct of his official affairs at each annual meeting, or on order of the Board of Directors; he shall pay out money only on order of the President of the Board of Directors, countersigned by the Recording Secretary.

SEC. 6. *The Board of Directors.* This Board shall constitute a Court for the trial of members for the violation of the Code of Ethics adopted by this Society, the Constitution and By-Laws, gross immorality or unprofessional conduct, or any other sufficient cause.

Charges against a member shall be made in writing, addressed to the President. He, on receiving them, shall call a meeting of the Board of Directors to investigate the same and the evidence thereof.

If a majority of the Board, after such investigation, shall be of opinion that the charges are well founded, they shall serve a copy of them on the accused, and appoint a day for hearing his defense, of which day he and the party making the charges, shall have at least ten days notice. If the accused in person or by counsel, who shall be an active member of this Society, having had a fair opportunity to hear the evidence against him, and to make his defense, shall be found guilty by a two-thirds vote of the Board, said Board shall affix and execute the penalty, subject to an appeal to the Society. If, after due notification, the accused party or counsel, shall fail to appear at the time and place of trial, without satisfactory excuse rendered at the time, he shall be considered as admitting the charges against him and shall be liable to sentence accordingly.

The Board shall furthermore manage the business part of the Society; take cognizance of the conduct of members, and perform the duties usually assigned to a Committee on Ethics.

SEC. 7. *Board of Examiners.* The Board of Examiners shall perform the duties as specifically prescribed by the Law of the State of Ohio.

ARTICLE II.

DUTIES AND PRIVILEGES OF MEMBERS.

SECTION 1. *Active Members*, shall on election, sign the Constitution, By-Laws and Code of Ethics, and they only shall have the right to vote and hold office.

SEC. 2. *Honorary Members*, shall be exempt from all payment of dues, and shall be entitled to all privileges of the floor at regular meetings.

ARTICLE III.

FEES AND ANNUAL DUES.

SECTION 1. The application for active membership must be accompanied by a fee of three dollars (which includes the first year's dues), and every active member shall pay an annual due of two dollars in advance, and no member subject to annual dues shall be permitted to speak on any subject until his dues are paid to the Treasurer.

Those in arrears for dues shall not be eligible for office, or vote on any subject before the Society; and in case the dues of any active member remain unpaid at the close of the second annual meeting thereafter, the name of such delinquent shall be stricken from the list of active members without further action.

SEC. 2. *Honorary Members*, are exempt from fees or dues.

ARTICLE IV.

No moneys of the Society shall be appropriated for any purpose, without the approval and recommendation of the Board of Directors, and then only by a vote of two-thirds of the members present; provided, however, that all current and necessary expenses of the Society may be passed by a majority vote of active members.

ARTICLE VI.

MEETINGS.

The annual meetings of the Ohio State Dental Society (as reorganized) shall be held on the last Wednesday of the month of October, at 10 o'clock a. m., continuing to final adjournment, at such place as the majority of its members may determine.

ARTICLE VII.

QUORUM

Seven members of the Board of Directors and five active members shall constitute a quorum at any annual meeting of the Society.

ARTICLE VIII.

ORDER OF BUSINESS.

1. Calling of the roll and payment of dues.
2. Reading of the records of the previous meeting.
3. Report of the Committee of Arrangements.
4. Reports of Standing Committees.
5. Reports of Special Committees.
6. Unfinished Business.
7. New Business.

ARTICLE IX.

RULES OF ORDER.

SECTION 1. No one shall be permitted to address the Society until he has been recognized by the Chair and his name pronounced; nor shall any one speak more than twice on any subject, nor longer than fifteen minutes in all, unless by consent.

SEC. 2. A member called to order while speaking, shall take his seat and the debate be suspended until the point of order is decided by the President.

SEC. 3. All questions of order shall be decided by the Chair, subject to an appeal, which shall be determined by a majority vote without debate.

SEC. 4. All resolutions and amendments shall be offered in writing.

SEC. 5. All papers presented to and read before the Society, become the property of the body; the Secretary shall be the responsible custodian thereof.

SEC. 6 Any member may call for a division of a question when the sense will admit of it.

SEC. 7 All questions not provided for by these rules, shall be determined by parlimentary usage, as set forth in "Cushing's Manual."

ARTICLE X.

All proposed changes in, or amendments to these By-Laws, shall be in writing, presented at a regular meeting, signed by five active members; and such alterations or amendments shall lie over until the next regular meeting, and not be acted upon until that time, and then adopted only by a three-fourths vote of the active members present.

The Rules and By Laws may be suspended for the time being by a unanimous vote.

The Constitution and By-Laws were then signed by the following gentlemen, the first eighteen being those who are the incorporators in the order in which they signed the Articles of Incorporation at Columbus, in October, 1884:

The following are the names of the active membess at the close of the meeting. viz. :

1. Geo. Watt.........................................Xenia.
2. C. H. James.................................Cincinnati.
3. C. H. Harroun.................................Toledo.
4. I. Williams*........................New Philadelphia.
5. J. Taft...........................................Cincinnati.
6. D. Gibbons*......................................Warren.
7. A. F. Emminger...............................Columbus.
8. E. G. Betty.....................................Cincinnati.
9. H. H. Harrison......................................Cadiz.
10. B. R. Taber..............................Bowling Green.
11. W. H. Sillito.......................................Xenia.
12. F. A. Hunter.................................Cincinnati.
13. H. A. Smith ................................ "
14. F. H. Rehwinkel.............................Chillicothe.
15. G. W. Keely.......................................Oxford.
16. A. Berry.........................................Cincinnati.
17. J. R. Callahan,................................Hillsboro.
18. C. R. Butler...................................Cleveland.

* Not present at this meeting.

19. D. R. Jennings................................Cleveland.
20. C. R. Dennis..................................Portsmouth.
21. Chas. Welch...................................Wilmington.
22. L. B. Welch...................................        "
23. M. H. Fletcher................................Cincinnati.
24. J. E. Robinson................................Cleveland.
25. F. W. Sage....................................Cincinnati.
26. J. Stephan....................................Cleveland.
27. A. Terry......................................Norwark.
28. J. S. Bason...................................Athens.
29. W. H. Hague...................................New Vienna.
30. F. C. Runyan..................................Springfield.
31. Henry Barnes..................................Cleveland.
32. W. D. Tremper.................................Portsmouth.
33. N. S. Hoff....................................Cincinnati.
34. Wm. Taft......................................        "
35. J. R. Safford.................................Gallipolis.
36. H. R. Clark...................................Circleville.
37. W. F. Galbreath...............................Greenfield.
38. John Galbreath................................Chillicothe.
39 L. J. Mitchell.................................Delaware.
40. W. R. Lilly...................................Circleville.
41. G. A. Dille...................................Athens.
42. W. H. Todd....................................Columbus.
43. E. J. Lilly...................................Circleville.

On motion, while the Articles were being signed, Dr. Keely, the Treasurer, reported a balance on hand of $41.14. Expenses of incorporation, $12.50, leaving a net balance of $28.64. Drs. Harroun and Harrison were appointed Auditing Committee, and found the accounts correct. On motion of Dr. Betty, Dr. Watt was transferred from the list of active members to that of honorary, at his own request, being the position he occupied before the re-organization. A motion was carried, that all members, incorporators and others, should pay a fee of $3.00 upon signing the Articles; this, in justice to all, and to equalize the payment of dues and initiation fee. New members were balloted for and signed as above. Adjourned.

